# Crystal structure of pyruvate decarboxylase from *Zymobacter palmae*

**DOI:** 10.1107/S2053230X16012012

**Published:** 2016-08-26

**Authors:** Lisa Buddrus, Emma S. V. Andrews, David J. Leak, Michael J. Danson, Vickery L. Arcus, Susan J. Crennell

**Affiliations:** a Department of Biology and Biochemistry, University of Bath, Claverton Down, Bath BA2 7AY, England; b School of Science, Faculty of Science and Engineering, University of Waikato, Private Bag 3105, Hamilton 3240, New Zealand

**Keywords:** *Zymobacter palmae*, pyruvate decarboxylase, lyase, crystal structure, TPP-dependent enzyme

## Abstract

Pyruvate decarboxylase (PDC; EC 4.1.1.1) is a thiamine pyrophosphate- and Mg^2+^ ion-dependent enzyme that catalyses the non-oxidative decarboxylation of pyruvate to acetaldehyde and carbon dioxide. It is rare in bacteria, but is a key enzyme in homofermentative metabolism, where ethanol is the major product. Here, the previously unreported crystal structure of the bacterial pyruvate decarboxylase from *Zymobacter palmae* is presented. The crystals were shown to diffract to 2.15 Å resolution. They belonged to space group *P*2_1_, with unit-cell parameters *a* = 204.56, *b* = 177.39, *c* = 244.55 Å and *R*_r.i.m._ = 0.175 (0.714 in the highest resolution bin). The structure was solved by molecular replacement using PDB entry 2vbi as a model and the final *R* values were *R*_work_ = 0.186 (0.271 in the highest resolution bin) and *R*_free_ = 0.220 (0.300 in the highest resolution bin). Each of the six tetramers is a dimer of dimers, with each monomer sharing its thiamine pyrophosphate across the dimer interface, and some contain ethylene glycol mimicking the substrate pyruvate in the active site. Comparison with other bacterial PDCs shows a correlation of higher thermostability with greater tetramer interface area and number of interactions.

## Abstract 2

The crystal structure of *Z. palmae* pyruvate decarboxylase was elucidated at 2.15 Å resolution.

## 1. Introduction   

Pyruvate decarboxylase (PDC; EC 4.1.1.1) catalyses the non-oxidative decarboxylation of pyruvate to acetaldehyde with the release of carbon dioxide, and is a key enzyme in homofermentative metabolism, where ethanol is the main fermentation product. Studies of *Zymomonas mobilis* PDC showed that its correct folding and activity strongly depend on the binding of the cofactors thiamine pyrophosphate (TPP) and Mg^2+^ ions (Pohl *et al.*, 1994 ▸).

*Zymobacter palmae*, which was originally isolated from palm sap (Okamoto *et al.*, 1993 ▸), is one of the few bacteria that employs a homofermentative metabolism. It is a Gram-negative, facultatively anaerobic mesophile that is able to utilize a variety of hexose sugars and oligosaccharides in producing ethanol (Horn *et al.*, 2000 ▸). This is the only member of the Halomonadaceae family that utilizes a PDC in their fermentation metabolism (de la Haba *et al.*, 2010 ▸).

PDCs are relatively widespread in plants and fungi, but are rarely found in bacteria. To date, only six bacterial PDCs have been described, including that found in *Z. palmae* (ZpPDC; PDB entry 5euj; this study). The *Z. mobilis* enzyme (ZmPDC) has been extensively studied, with a variety of structural variants published [PDB entries 1zpd (Dobritzsch *et al.*, 1998 ▸), 2wva, 2wvg and 2wvh (Pei *et al.*, 2010 ▸), 3oei (Meyer *et al.*, 2010 ▸) and 4zp1 (Wechsler *et al.*, 2015 ▸)]. Other bacterial PDCs include those from *Acetobacter pasteurianus* (ApPDC; PDB entry 2vbi; D. Gocke, C. L. Berthold, G. Schneider & M. Pohl, unpublished work), *Gluconoacetobacter diazotrophicus* (GdPDC; PDB entry 4cok; van Zyl, Schubert *et al.*, 2014 ▸) and *Gluconobacter oxydans* (GoPDC; van Zyl, Taylor *et al.*, 2014 ▸), and from the only known Gram-positive species possessing a PDC, *Sarcina ventriculi* (SvPDC; Lowe & Zeikus, 1992 ▸).

**Figure 1:**
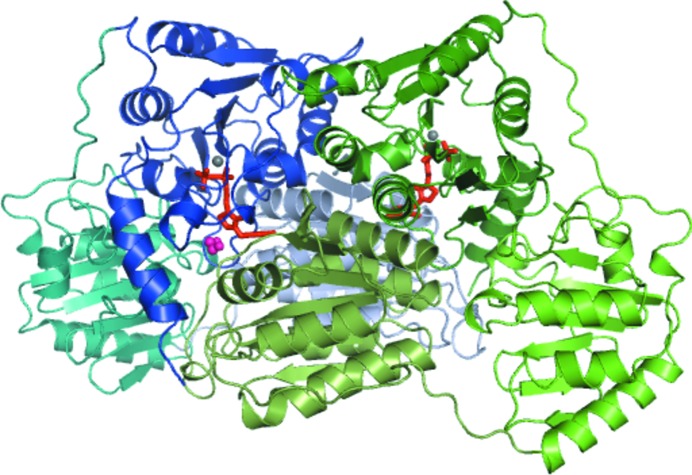
Cartoon representation of the ZpPDC dimer. One monomer is coloured blue, with the PYR domain (residues 1–190) in pale blue, the R domain (residues 191–355) in teal and the PP domain (residues 356–556) in dark blue. The second monomer is coloured green, with the PYR domain in pale green, the R domain in bright green and the PP domain in dark green. The active-site magnesium ions are represented as grey spheres and an 1,2-ethanediol molecule bound in the active site of the blue monomer as pink spheres. Two TPP molecules bound between the PYR domain of one monomer and the PP domain of the other nonomer are represented as orange sticks.

ZpPDC forms a tetramer from four identical subunits in a dimer-of-dimers fashion. Each subunit consists of 556 amino acids with a molecular mass of 59.4 kDa. Each monomer contains a pyrimidine-binding (PYR) domain, a regulatory (R) domain and a pyrophosphate-binding (PP) domain. The TPP molecules bind across both subunits in each dimer, with the pyrophosphate group binding to the PP domain from one subunit and the pyrimidine ring binding to the PYR domain from the second subunit, thus forming two active sites in the dimer (Fig. 1 ▸).

Unlike yeast PDCs, bacterial PDCs are not allosterically activated, with the exception of SvPDC (Raj *et al.*, 2002 ▸). In the first step of the catalytic cycle, TPP is protonated at N-1′ and deprotonated at 4′-NH_2_. This imino tautomer in turn promotes deprotonation at C2 on the thiazolium ring, thus creating the active ylid. The nucleophilic attack of the ylid on the carbonyl group of pyruvate generates a lactyl adduct (C2-α-lactylthiamine diphosphate intermediate), decarboxylation of which yields the enamine intermediate with concomitant release of carbon dioxide. This intermediate is then protonated, producing hydroxyethyl TPP, and finally the release of acetaldehyde regenerates the ylid (Pei *et al.*, 2010 ▸; van Zyl, Schubert *et al.*, 2014 ▸).

Bacterial PDCs are of great interest for biotechnological applications, in particular for bioethanol production as a second-generation renewable transport fuel. ZmPDC has thus been successfully used to create mesophilic ethanologenic strains of *Escherichia coli* (Ingram *et al.*, 1987 ▸), *Klebsiella oxytoca* (Ingram *et al.*, 1999 ▸) and *Bacillus* spp. (Barbosa & Ingram, 1994 ▸). Furthermore, ZpPDC has been functionally expressed in *Lactococcus lactis* (Liu *et al.*, 2005 ▸). However, this is by no means an exhaustive list. These earlier studies in mesophilic organisms seemed promising, and in recent years growing interest has developed in utilizing bacterial PDCs for high-temperature ethanol-production processes in thermophilic bacteria. Attempts have been made to express ZmPDC (Thompson *et al.*, 2008 ▸), ZpPDC (Taylor *et al.*, 2008 ▸) and GoPDC (van Zyl, Taylor *et al.*, 2014 ▸) in the thermophile *Geobacillus thermoglucosidasius*, with limited success.

In this study, we present the crystal structure of recombinant ZpPDC at 2.15 Å resolution together with a comparison to the other known bacterial PDCs, thereby increasing the structural information available on these rare enzymes. Thus, we support the quest to understand bacterial PDCs and ongoing protein-engineering efforts.

## 2. Materials and methods   

### 2.1. Macromolecule production   

The gene encoding ZpPDC (annotated in GenBank as AF474145.1) was PCR-amplified from *Z. palmae* T109 genomic DNA, and included the introduction of PciI and XhoI restriction sites for cloning into pET-28a(+) (Novagen). The resulting construct adds a thrombin cleavage site, a 3×Gly linker and a hexahistidine tag to the C-terminus of the ZpPDC, and was transformed into *E. coli* BL21(DE3) cells for expression.

For overexpression, the *E. coli* cells were grown in autoinducing Overnight Express TB (Novagen) medium supplemented with 100 µg ml^−1^ kanamycin and 5 m*M* thiamine chloride at 303 K for 16 h with shaking at 220 rev min^−1^.

The cells were harvested by centrifugation (4000*g*, 277 K) and resuspended in His-bind buffer (20 m*M* Tris pH 8, 300 m*M* NaCl, 20 m*M* imidazole). The cells were lysed by sonication on ice and the insoluble debris was removed by centrifugation (17 000*g*, 277 K, 30 min). The supernatant was loaded onto a HisTrap HP (GE Healthcare) 5 ml column for nickel-affinity chromatography. The column was washed with at least ten column volumes of His-bind buffer before eluting the protein with increasing concentrations of His-elute buffer (20 m*M* Tris pH 8, 300 m*M* NaCl, 1 *M* imidazole) on an ÄKTAexplorer FPLC system (GE Healthcare), monitoring the eluted protein at 280 nm.

The eluted protein was buffer-exchanged into 50 m*M* 2-(*N*-morpholino)ethanesulfonic acid (MES) pH 6.5, 20 m*M* MgSO_4_, 3 m*M* TPP using a Superdex 200 10/300 GL gel-filtration column (GE Healthcare) on the ÄKTAexplorer FPLC system and was purified to >95% homogeneity as determined by 12% SDS–PAGE analysis.

Protein-activity assays were performed as described in Raj *et al.* (2002 ▸) with the reaction mixture consisting of 0.15 m*M* NADH, 20 m*M* MgSO_4_, 3 m*M* TPP, 29 m*M* pyruvate, 10 U *Saccharomyces cerevisiae* alcohol dehydrogenase (Sigma–Aldrich) in 50 m*M* MES pH 6.5 at 303 K. The temperature optimum was determined as described by Gocke *et al.* (2009 ▸) following the depletion of pyruvate at 320 nm.

Information relating to the production of recombinant ZpPDC is summarized in Table 1 ▸.

### 2.2. Crystallization   

Crystals of the purified ZpPDC were obtained using the hanging-drop vapour-diffusion method at 291 K. The purified ZpPDC was incubated with 2 m*M* pyruvate for 30 min at room temperature (293 K), and 2 µl drops of the enzyme/pyruvate solution mixed with crystallization solution [0.15 *M* sodium citrate pH 5.5, 14%(*w*/*v*) PEG 3350] in a 1:1 ratio were placed onto cover slips and equilibrated against 400 µl reservoir solution. Crystals were looped-out and soaked in cryoprotectant [10%(*w*/*v*) glycerol added to the crystallization buffer] before flash-cooling and storage in liquid nitrogen. Crystallization information is summarized in Table 2 ▸.

### 2.3. Data collection and processing   

X-ray diffraction data were collected to 2.15 Å resolution on beamline MX2 at the Australian Synchrotron (AS) in Melbourne, Australia. *AIMLESS* (Evans & Murshudov, 2013 ▸), *iMosflm* (Battye *et al.*, 2011 ▸) and *BALBES* (Long *et al.*, 2008 ▸) were used for data scaling, data reduction and phasing, respectively. The crystal belonged to space group *P*2_1_, with unit-cell parameters *a* = 204.56, *b* = 177.39, *c* = 244.55 Å, and contained six tetramers in the asymmetric unit. Data-collection and processing statistics are summarized in Table 3 ▸.

### 2.4. Structure solution and refinement   

The structure was solved by molecular replacement using ApPDC (PDB entry 2vbi; 76% amino-acid identity) as the starting model. The structure was refined by iterative cycles of manual building and modelling in *Coot* (Emsley *et al.*, 2010 ▸) and refinement in *REFMAC*5 (*CCP*4 suite; Winn *et al.*, 2001 ▸; Potterton *et al.*, 2003 ▸; Murshudov *et al.*, 2011 ▸). Initially, the noncrystallographic symmetry was used to minimize the rebuilding task; however, in the later stages this was not used and all 24 monomers were refined independently. The quality of the final model was checked using *MolProbity* (Chen *et al.*, 2010 ▸). The structure has been submitted to the Protein Data Bank and assigned PDB code 5euj. Table 4 ▸ summarizes the structure-solution and refinement statistics.

## 3. Results and discussion   

### 3.1. Overall structure   

The crystal structure of ZpPDC was determined by molecular replacement using ApPDC (PDB entry 2vbi) as a search model, with the correct solution identified by the presence of electron density for TPP. As described for other bacterial PDCs, the quaternary structure of ZpPDC is a homotetramer, or dimer of dimers. The domains can be assigned as follows: amino acids 1–190, PYR (pyrimidine-binding domain, with 176–190 being a linker); 191–355, R (regulatory domain, with 345–355 being a linker); 356–555, PP (pyrophosphate-binding domain) (Fig. 1 ▸). It should be noted that the amino-acid sequence differs from the GenBank entry (AAM49566.1) in two places. The GenBank entry erroneously contains Arg134 and Glu245; both are actually Ala as shown by both our resequencing of the gene and the observed electron density.

The final model contains six tetramers in the asymmetric unit, each with a surface area of 65 000 Å^2^. It comprises 24 amino-acid chains of 555 amino acids, each with a TPP and a Mg^2+^ ion, and 4147 water molecules. Comparison of all 24 monomers yielded an average root-mean-square deviation (r.m.s.d.) of 0.173 Å, indicating that the structures are similar. Despite crystallization in the presence of pyruvate, no pyruvate molecules were visible anywhere in the structure, probably owing to catalytic turnover of the substrate to acetaldehyde and subsequent loss of this volatile product. However, six 1,2-ethanediol (EDO) molecules (the PEG monomer) are present, four of which can be found in the active sites in chains *I*, *N*, *Q* and *R*.

**Figure 2:**
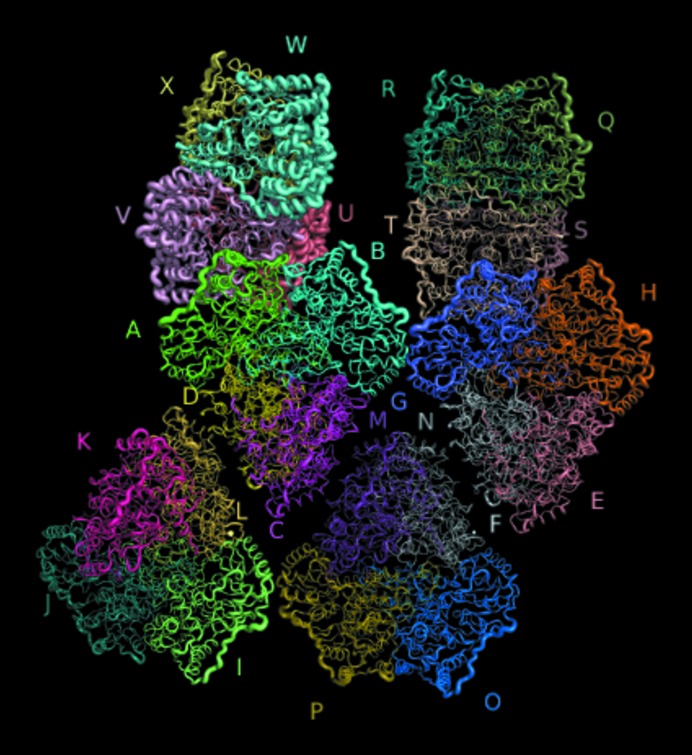
C^α^ representation of the asymmetric unit containing the six tetramers coloured by chain and labelled with the chain name, with the thickness of the trace determined by the *B* factors. The *UVWX* tetramer has anomalously high *B* factors and correspondingly poor electron density.

Overall, the electron-density map was of good quality, apart from chains *U*, *V*, *W* and *X*, which seem exceptionally flexible (Fig. 2 ▸). The average *B* factor for all tetramers is 27 Å^2^; the average *B* factor for *UVWX* is 42 Å^2^ and the *B* factors of the individual monomers are also considerably higher within this tetramer (Table 4 ▸). Other common flexible areas include the exposed N-terminal Met, the exposed C-terminus and the 340–355 loop, which is the exposed linker between the R and PP domains.

The final model was refined to an *R* factor of 18.6% and an *R*_free_ value of 22.3%, using data between 75.40 and 2.15 Å resolution. 98.05% of the residues are located in favoured regions of the Ramachandran plot. In chains *G*, *J*, *K*, *M* and *O*, Ser73 is located in the disallowed region, but is well defined in its electron density and seems to be located in a tight bend. This has been noted previously in ZmPDC (Dobritzsch *et al.*, 1998 ▸).

### 3.2. Functional and structural comparison   

Most bacterial PDCs studied so far are very similar in their amino-acid sequences and kinetic parameters, with the exception of SvPDC. It is thought that SvPDC is more closely related to fungal PDCs, whereas the other bacterial PDCs are more similar to plant PDCs (Raj *et al.*, 2002 ▸). SvPDC is the only PDC to be identified from a Gram-positive bacterium, shares only 31% amino-acid identity with ZpPDC and shows sigmoidal rather than Michaelis–Menten kinetics.

ZpPDC shows a high temperature optimum at 65°C, and retains 80% activity after incubation at 65°C for 30 min (Raj *et al.*, 2002 ▸; confirmed in this study), making it one of the most thermostable bacterial PDCs currently known. See Table 5 ▸ for a summary of the properties of known PDCs.

Based on the r.m.s.d. values, the tetramers in PDB entry 5euj are more similar to each other than to other known bacterial PDCs. The *MNOP* tetramer is the most representative of the six tetramers in PDB entry 5euj, and was used to compare the ZpPDC structure with other known bacterial PDC structures.

The residues involved in interactions on interfaces (*i.e.* forming hydrogen bonds or salt bridges) are well conserved overall in all bacterial PDCs analysed. Table 6 ▸ summarizes the interface areas, and Table 7 ▸ summarizes the numbers of interactions made between different interfaces. A trend of increasing interface area and increasing number of salt bridges can be observed as the thermoactivity and thermostability of the PDCs increase.

Conserved regions were analysed by using *PROMALS*3*D* (Pei *et al.*, 2008 ▸) to generate a structure-based alignment. The PYR and PP domains are well conserved and the R domain and the linker regions much less so. PDB entry 1zpd contains some extra residues in the R and PP domains, as discussed in van Zyl, Schubert *et al.* (2014 ▸). However, none of these factors seem to correlate with the thermostability and thermoactivity differences that are observed between these PDCs.

### 3.3. The active site and TPP binding   

There is good-quality electron-density evidence for a TPP molecule in each of the 24 chains of PDB entry 5euj. However, as noted previously (Dobritzsch *et al.*, 1998 ▸), the bound TPP appears to be chemically modified at C2. The electron-density evidence suggests that the thiazolium ring has been opened and the C2 atom has been lost, as seen in PDB entry 1zpd. This was suggested to be most likely owing to partial degradation of the TPP during crystallization (Dobritzsch *et al.*, 1998 ▸).

Through pseudo-222 symmetry, the four monomers in a tetramer create four cofactor- and substrate-binding sites located in narrow clefts on the interfaces between the PYR domain of one monomer and the PP domain of a second monomer. The TPP molecule binds as indicated by the domain nomenclature (Fig. 1 ▸), with the pyrophosphate group binding to the PP domain of one monomer and the pyrimidine ring binding to the PYR domain of a second monomer in the dimer.

**Figure 3:**
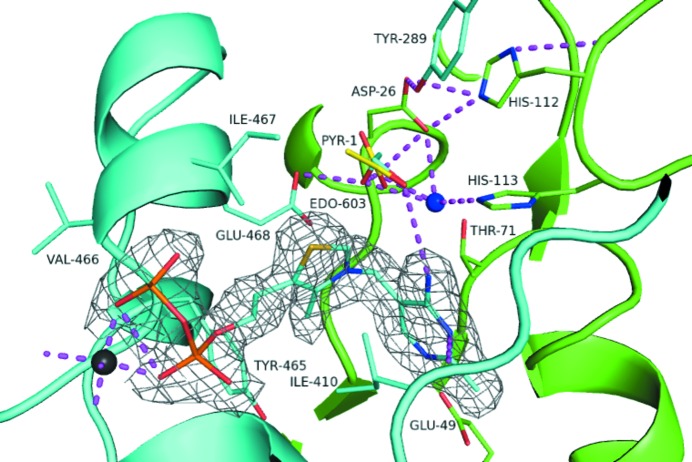
Cartoon and stick depiction of the active site. Residues of one monomer are coloured cyan; residues of the other monomer are coloured green. The magnesium ion (dark grey) and water molecules (blue) are represented as spheres. The 1,2-ethanediol (EDO) and TPP of the cyan chain are shown as stick models and are coloured by atom. Pyruvate (PYR, yellow) has been overlaid following superposition of *Z. mobilis* PDC (PDB entry 2wva, chain *F*) and lies on top of the EDO. The 2*F*_o_ − *F*_c_ density (grey) surrounding the TPP is shown contoured at 1σ.

Ile410 from the first monomer holds TPP in a V-shape (Fig. 3 ▸). The backbone amide groups of Ile467 and Val466 form hydrogen bonds to the O atoms of the pyrophosphate. Glu49 (from the second monomer) forms a hydrogen bond to the N1 atom on the pyrimidine ring. This is essential for the C2 deprotonation mechanism (Pei *et al.*, 2010 ▸). Other residues involved in TPP binding are Tyr465 and Glu468 from one monomer around the pyrophosphate and Thr71 from the second monomer around the pyrimidine ring.

A water molecule supports the active-site arrangement, interacting with Asp26, Thr71 and His113, and is present even in the absence of substrate. This is thought to play a pivotal role in the organization of the substrate complex and of the hydrogen-bond network (Pei *et al.*, 2010 ▸).

The pyrophosphate group of TPP is anchored to the protein by a Mg^2+^ ion. It forms an octahedral coordination sphere with the two O atoms on the diphosphate group of the TPP, the side-chain O atoms of Asp435 and Asn462, the main-chain O atom of Gly464 and a water molecule.

**Figure 4:**
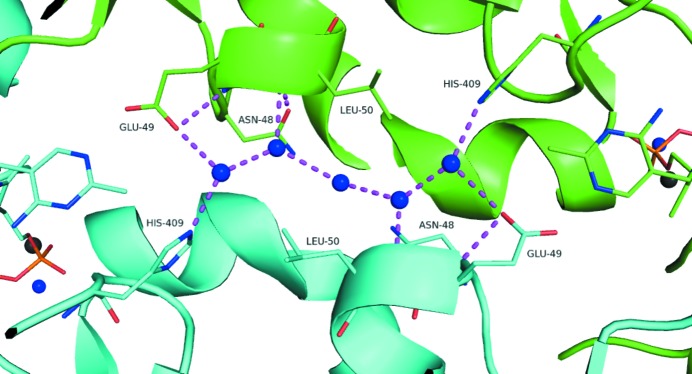
Model of the water tunnel connecting the two active sites in the ZpPDC dimer. Water molecules are shown as blue spheres and magnesium ions as dark grey spheres. Residues and the TPP from one monomer are shown as cyan (both C atoms and cartoon), while the other monomer in the dimer is coloured green.

The active sites appear to be connected by a water tunnel (Fig. 4 ▸). This has been noted in PDCs and other TPP-binding enzymes (Pei *et al.*, 2010 ▸; Frank *et al.*, 2004 ▸). Pei *et al.* (2010 ▸) suggested that it may play a role as a form of communication system between the active sites, perhaps as a proton-relay system. PDB entry 5euj shows the same pattern of water molecules as PDB entry 2wva. Similarly, the residues lining the tunnel seem to be well conserved and include Glu49, Asn48, Leu50 and His409.

A comparison of apo and holo ZmPDC structures by Pei *et al.* (2010 ▸) revealed that TPP binding induces a conformational change involving the loop and the adjacent α-helix between Asn467 and Tyr481 (Asn462 and Tyr476 in PDB entry 5euj). This region is structured in the apoenzyme and thus requires major conformational changes to accommodate TPP binding (Pei *et al.*, 2010 ▸). Similar changes would presumably occur in ZpPDC as this region is structurally highly conserved in bacterial PDCs.

### 3.4. Substrate binding and the catalysis mechanism   

As mentioned above, no pyruvate was found in the crystal structure presented here. However, EDO was bound in the active site of some chains, and aligns very well with the positioning of pyruvate when superposed with chain *F* of PDB entry 2wva (Fig. 3 ▸). This allowed the observation of inter­actions that might be made with pyruvate and comparison with those reported by Pei *et al.* (2010 ▸) and Dobritzsch *et al.* (1998 ▸).

Glu468 and Tyr289 from one monomer and Asp26, His112 and His113 from a second monomer interact with EDO through an extensive hydrogen-bond network.

Glu468 is very likely to play a key role in catalysis and is thought to be the proton acceptor from the 4′-NH_2_ group of TPP, thus deprotonating C2 to form the active ylid (Kern *et al.*, 1997 ▸; Pei *et al.*, 2010 ▸). It is further thought to form a stabilizing hydrogen-bond interaction with the dianion formed after the nucleophilic attack of the thiazolium-ring carbanion on pyruvate (Dobritzsch *et al.*, 1998 ▸). In the lactyl-TPP intermediate, the five-membered ring is positively charged, facilitating a reverse proton transfer from Glu468 to 4′-N. The now negatively charged Glu468 destabilizes the adjacent carboxylate group on the pyruvate and thus facilitates subsequent decarboxylation (Pei *et al.*, 2010 ▸). Tittmann *et al.* (2003 ▸) and Meyer *et al.* (2010 ▸) investigated the role of this residue further, and using a variety of mutants found it to be crucial in substrate binding and catalysis. His112 may not be directly involved in catalysis, but plays an important role in maintaining the active-site environment, in particular in retaining His113 uncharged. This in turn is essential to allow proton abstraction from C2 in the first step of catalysis (Dobritzsch *et al.*, 1998 ▸). Furthermore, His112 is likely to be involved in holding the carboxylate group of Asp26 in the correct state and position, supported by Tyr289 (Pei *et al.*, 2010 ▸). Asp26 may also be involved in acetaldehyde release (Pei *et al.*, 2010 ▸). His113 interacts with O3 of pyruvate and N4′ of TPP (Dobritzsch *et al.*, 1998 ▸).

Dobritzsch *et al.* (1998 ▸) remarked that large conformational changes upon substrate binding are unlikely owing to the extensive interface regions. Instead, it is thought that the C-terminal helix swings out of the way to allow access to the active site and closes upon substrate binding to create a hydrophobic active-site environment. This helix is also exposed in PDB entry 5euj, so it is likely that a similar mechanism applies here.

In summary, we present the crystal structure of *Z. palmae* PDC and a brief functional and structural comparison to known bacterial PDCs. They are structurally well conserved, which may allow the in-depth studies carried out on ZmPDC of the mechanism of folding as presented by Pohl *et al.* (1994 ▸) and the mechanism of catalysis as described by Dobritzsch *et al.* (1998 ▸), Pei *et al.* (2010 ▸) and Meyer *et al.* (2010 ▸) to be applied to ZpPDC. Structural analysis suggests that the different thermostability and thermoactivity displayed by these PDCs may be correlated with increased oligomeric interface and salt bridges, as has been seen in many other protein families (Sterner & Liebl, 2001 ▸). We hereby add to the structural knowledge of bacterial PDCs, generating information that has the potential to be very useful in design approaches for enzyme-engineering and biotechnological applications.

## Supplementary Material

PDB reference: pyruvate decarboxylase, 5euj

## Figures and Tables

**Table 1:** Details relating to the production of recombinant ZpPDC

Source organism	*Z. palmae* strain T109
DNA source	*Z. palmae* strain T109 genomic DNA
Forward primer†	5′-TAATACATGTATACCGTTGGTATGTACTTGGCAG-3′
Reverse primer‡	5′-GTGTACTCGAGGCCGCCGCCGCTGCCGCG-3′
Cloning vector	pET-28a(+) (Novagen)
Expression vector	pET-28a(+) (Novagen)
Expression host	*E. coli* BL21 (DE3)
Complete amino-acid sequence of the construct produced§	MYTVGMYLAERLAQIGLKHHFAVAGDYNLVLLDQLLLNKDMEQVYCCNELNCGFSAEGYARARGAAAAIVTFSVGAISAMNAIGGAYAENLPVILISGSPNTNDYGTGHILHHTIGTTDYNYQLEMVKHVTCAAESIVSAEEAPAKIDHVIRTALRERKPAYLEIACNVAGAECVRPGPINSLLRELEVDQTSVTAAVDAAVEWLQDRQNVVMLVGSKLRAAAAEKQAVALADRLGCAVTIMAAAKGFFPEDHPNFRGLYWGEVSSEGAQELVENADAILCLAPVFNDYATVGWNSWPKGDNVMVMDTDRVTFAGQSFEGLSLSTFAAALAEKAPSRPATTQGTQAPVLGIEAAEPNAPLTNDEMTRQIQSLITSDTTLTAETGDSWFNASRMPIPGGARVELEMQWGHIGWSVPSAFGNAVGSPERRHIMMVGDGSFQLTAQEVAQMIRYEIPVIIFLINNRGYVIEIAIHDGPYNYIKNWNYAGLIDVFNDEDGHGLGLKASTGAELEGAIKKALDNRRGPTLIECNIAQDDCTETLIAWGKRVAATNSRKPQALVPRGSGGGLEHHHHHH

† The PciI site for cloning is underlined.

‡ The XhoI site for cloning is underlined.

§ The C-terminal tag containing a thrombin cleavage site, a 3×Gly linker and a hexa-His tag is underlined.

**Table 2:** Crystallization conditions

Method	Hanging-drop vapour diffusion
Plate type	24-well
Temperature (K)	291
Protein concentration (mg ml^−1^)	4.8
Buffer composition of protein solution	50 m*M* MES pH 6.5, 20 m*M* MgSO_4_, 3 m*M* TPP
Composition of reservoir solution	0.15 *M* sodium citrate pH 5.5, 14%(*w*/*v*) PEG 3350
Volume and ratio of drop	2 µl, 1:1
Volume of reservoir (ml)	0.4

**Table 3:** Data collection and processing Two data collections, with different crystal-to-detector distances and rotation ranges, were combined to form the final data set. Values in parentheses are for the outer resolution shell.

Diffraction source	Beamline MX2, AS
Wavelength (Å)	0.9537
Temperature (K)	100.0
Detector	ADSC Q315r CCD
Crystal-to-detector distance (mm)	400/300
Rotation range per image (°)	0.5/0.25
Total rotation range (°)	180
Exposure time per image (s)	1
Space group	*P*2_1_
*a*, *b*, *c* (Å)	204.56, 177.39, 244.55
α, β, γ (°)	90, 112.94, 90
Mosaicity (°)	0.3
Resolution range (Å)	75.470–2.150 (2.190–2.150)
Total No. of reflections	5108329 (153406)
No. of unique reflections	858032 (41628)
Completeness (%)	98.9 (96.9)
Multiplicity	6.0 (3.7)
〈*I*/σ(*I*)〉	19.9 (2.3)
*R*_r.i.m._ †	0.175 (0.714)
Overall *B* factor from Wilson plot (Å^2^)	19.9

† Estimated *R*_r.i.m._ = *R*_merge_[*N*/(*N* − 1)]^1/2^, where *N* is the data multiplicity.

**Table 4:** Structure solution and refinement Values in parentheses are for the outer shell.

Resolution range (Å)	225.21–2.15 (2.207–2.151)
Completeness (%)	98.8 (97.6)
σ Cutoff	*F* > 0.000σ(*F*)
No. of reflections, working set	814954 (59398)
No. of reflections, test set	42940 (3176)
Final *R*_cryst_	0.186 (0.271)
Final *R*_free_	0.220 (0.300)
Cruickshank DPI	0.2148
No. of non-H atoms	
Protein	102016
Ligand	648
Solvent	4316
Total	107004
R.m.s. deviations	
Bonds (Å)	0.008
Angles (°)	1.307
Average *B* factors (Å^2^)	
Protein	26.935†
Ligand	24.626
Ramachandran plot	
Most favoured (%)	98.05
Allowed (%)	1.91

† Average *B* factors per chain (Å^2^): *A*, 28.241; *B*, 27.376; *C*, 21.301; *D*, 22.281; *E*, 21.252; *F*, 18.55; *G*, 25.637; *H*, 27.299; *I*, 25.189; *J*, 22.729; *K*, 24.382; *L*, 23.331; *M*, 18.054; *N*, 18.722; *O*, 24.315; *P*, 23.331; *Q*, 27.589; *R*, 27.29; *S*, 25.579; *T*, 24.993; *U*, 44.654; *V*, 43.492; *W*, 47.915; *X*, 32.927.

**Table 5:** Properties of known bacterial PDCs in order of decreasing thermostability

	ZpPDC	ApPDC	ZmPDC	GdPDC	GoPDC	SvPDC
Gram status	Negative	Negative	Negative	Negative	Negative	Positive
Amino-acid identity (%)	Reference	73	63	71	67	31
Temperature optimum (°C)	65	65†	60†	45–50‡	53§	NA
Temperature dependence of activity retention	60°C, 100%	50°C, 100%	45°C, 85%	NA (half-life at 60°C, 0.3 h‡)	55°C, 98%	45°C, 95%
	65°C, 80%†	60°C, 65%	60°C, 65%		60°C, 70%	50°C, 0%¶
	70°C, 0%	65°C, 45%	65°C, 45%		65°C, 40%§	
		70°C, 5%¶	70°C, 0%¶			
Kinetics	Michaelis–Menten	Michaelis–Menten	Michaelis–Menten	Michaelis–Menten	Michaelis–Menten	Sigmoidal
*V*_max_ (U mg^−1^)	165 ± 3 (pH 6.5)	110 ± 2 (pH 6.5)†	121 (pH 6.5)†	20 (pH 5)	57 (pH 5)	103[Table-fn tfn10]
	116 ± 2 (pH 6.5)†	97 (pH 5)¶	100 (pH 6)¶	39 (pH 6)	47 (pH 6)	45 (pH 6.5)¶
	130 (pH 6)¶	79 (pH 7)¶	78 (pH 7)¶	43 (pH 7)‡	125 (pH 7)§	35 (pH 7)¶
	140 (pH 7)¶		120[Table-fn tfn11]			
			181†			
*K*_m_ (S_0.5_) (m*M*)	0.67 ± 0.05 (pH 6.5)	2.8 ± 0.2 (pH 6.5)†	1.3 (pH 6.5)†	0.06 (pH 5)	0.12 (pH 5)	13[Table-fn tfn10]
	2.5 ± 0.2 (pH 6.5)†	0.39 (pH 5)¶	0.43 (pH 6)¶	0.6 (pH 6)	1.2 (pH 6)	5.7 (pH 6.5)
	0.24 (pH 6)¶	5.1 (pH 7)¶	0.94 (pH 7)¶	1.2 (pH 7) ‡	2.8 (pH 7)§	4.0 (pH 7)¶
	0.71 (pH 7)¶		0.31 (pH 6)‡			
			1.1[Table-fn tfn11]			
			0.4 (pH 6)†			
PDB entry	5euj	2vbi	1zpd	4cok	NA	NA
GenBank gene	AF474145	AF368435.1	M15393.2	KJ746104.1	KF650839.1	AAL18557.1
GenBank protein	AAM49566.1	AAM21208.1	AAA27696.2	AIG13066.1	AHB37781.1	AF354297.1
R.m.s.d.	Reference	0.70	0.70	0.62	NA	NA
*Q* scores	Reference	0.94	0.90	0.94	NA	NA

† Gocke *et al.* (2009 ▸).

‡ van Zyl, Schubert *et al.* (2014 ▸).

§ van Zyl, Taylor *et al.* (2014 ▸).

¶ Raj *et al.* (2002 ▸).

†† Lowe & Zeikus (1992 ▸).

‡‡ Siegert *et al.* (2005 ▸).

§§ Bringer-Meyer *et al.* (1986 ▸).

¶¶ Meyer *et al.* (2010 ▸).

**Table 6:** Comparison of interface areas of known bacterial PDCs in order of decreasing thermostability

	ZpPDC (PDB entry 5euj)	ApPDC (PDB entry 2vbi)	ZmPDC (PDB entry 1zpd)	GdPDC (PDB entry 4cok)
Interface area between monomers within a functional dimer (Å^2^)	3813.13	3761.3	4144.5 (4387†)	3749.8
Percentage of total surface of the monomer	17.08	16.95	18.39 (19.4†)	17.83
Interaction area between two functional dimers (Å^2^)	2912.16	2840	2489.2 (4405†)	1851.4
Percentage of total surface of one dimer	13.06	12.78	11.03 (12.1†)	8.79

† Dobritzsch *et al.* (1998 ▸).

**Table 7:** Comparison of interactions within interfaces of bacterial PDC structures in order of decreasing thermostability Interactions were determined using *PISA*. Neighbour, interactions between neighbouring monomers; diagonal, interactions between monomers diagonally across tetrameric centre.

	ZpPDC (PDB entry 5euj)		ApPDC (PDB entry 2vbi)		ZmPDC (PDB entry 1zpd)		GdPDC (PDB entry 4cok)	
	Hydrogen bonds	Salt bridges	Hydrogen bonds	Salt bridges	Hydrogen bonds	Salt bridges	Hydrogen bonds	Salt bridges
Dimer interface	73	12	61	16	76 (66†)	14 (7†)	63	13
Major tetramer interface (neighbour)	31	24	34	14	29 (64†)	8 (25†)	17	9
Minor tetramer interface (diagonal)	2	0	4	0	6	2	4	3
TPP pyrimidine ring	11	0	10	0	12	0	10	0

† Dobritzsch *et al.* (1998 ▸).
